# Mini-laparotomic Colpotomy for a Cervicovaginal Leiomyoma: Preservation of hymenal integrity

**Published:** 2016-03

**Authors:** Ibrahim Yalçin, Emre Pabuçcu, Korhan kahraman, Murat Sönmezer

**Affiliations:** *Department of Obstetrics and Gynecology, School of Medicine, Ankara University, Ankara, Turkey.*

**Keywords:** *Colpotomy*, *Hymen*, *Minilaparotomy*, *Leiomyoma*

## Abstract

**Background:**

Leiomyomas are the most common benign tumors of the uterus. Removal of the prolapsed pedunculated submucous myoma represents a distinct entity. Evaluation and treatment of such cases may need intervention via the hymen.Mini-laparotomic management of a pedunculated submucous myoma while preserving hymen integrity in a virginal patient is described as a safe alternative..

**Case:**

A 30-year old, nulliparous virgin woman admitted to the outpatient- clinic with the complaint of irregular menstrual bleeding ongoing for three months. Pelvic ultrasound revealed a 5×6 cm solid mass in the cervico-vaginal location that filled the vaginal margins. Due to the patient’s consistent desire for preserving hymenal integrity, mini-laparotomic colpotomy was performed and the mass was removed successfully.

**Conclusion:**

Mini-laparotomic colpotomy, preserving hymen integrity, provides excellent visualization and it is a convenient and effective tool in the management of a cervicovaginal pedunculated submucous myoma.

## Introduction

Leiomyomas are the most common benign tumors of the uterus and the female genital tract. According to its localization, they are classified as submucosal, intramural, or subserosal. Some of submucosal myomas may be pedunculated and eventually can protrude through the cervical canal or even into the vagina. These myomas generally become necrotic and infected as a result of inadequate blood supply through the pedicle (1). Removal of prolapsed pedunculated submucous myomas represents a distinct entity. Virginity, defined as an intact hymen, is considered as a sign of sexual purity in some societies and therefore, anatomic integrity of hymen accounts for one of major social concerns. Vaginal surgical procedures in patients with intact hymenal ring obviously come with some concerns for both patient and the clinician. 

In this case report, management of a prolapsed pedunculated submucous leiomyoma will discussed in virgin patient.

## Case report

30-year old, nulliparous virgin Turkish Muslim woman admitted to Ankara University Department of Obstetrics and Gynecology outpatient-clinic in May 2012 with complaint of irregular menstrual bleeding ongoing for 3 months. Other symptoms included pelvic pain and yellow-pinkish vaginal discharge. Her medical history was uneventful and the patient denied any sexual intercourse. 

On gynecologic examination, vulvar and vaginal inspection was uneventful with annular intact hymen. Abdominal examination was unremarkable, however rectal examination revealed significant vaginal fullness. Her laboratory tests, including tumor markers of CA-125, carsinoembryonic antigen (CEA), CA-19-9 and alpha- fetoprotein (AFP) were in normal ranges despite severe anemia as her hemoglobin was 7.1 g/dl. Pelvic ultrasound revealed a 5×6 cm solid mass in the cervico-vaginal location which filled the vaginal margins. Mass was not belonging through endometrial cavity. Uterus and adnexa were considered normal. 

Surgical removal of the myoma via vaginal approach was offered to patient as a first line surgical option. Patient was informed about written medical report that the integrity of hymen can be lost during medical intervention and hymenal repair can performed during the procedure. Because of very strict and standpatter nature of patient and her family, she was denied to have vaginal approach. Due to patient’s consistent desire for preserving hymenal integrity, written consent was obtained from the patient for abdominal approach and mini-laparotomy was performed. 

**Figure 1:**
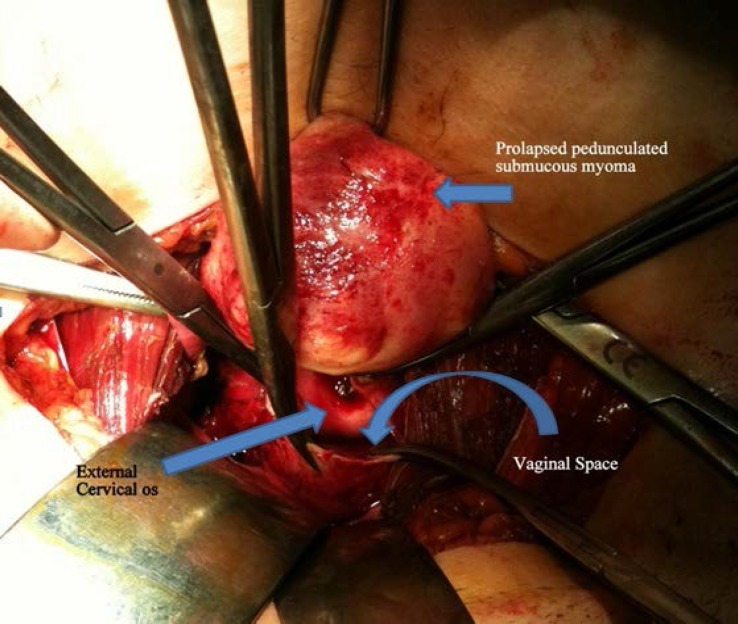
Laparotomic colpotomy. Submucous myoma was prolapsed from cervix

During inspection; uterus, adnexa and pelvic surfaces were considered normal. Bulky appearance that located under the anterior wall of the vagina was observed and palpated. The bladder was then liberated and a small incision was performed on the anterior vaginal wall. A firm regular pedunculated mass (Figure 1) was identified and delivered with rotational movements. 

**Figure 2:**
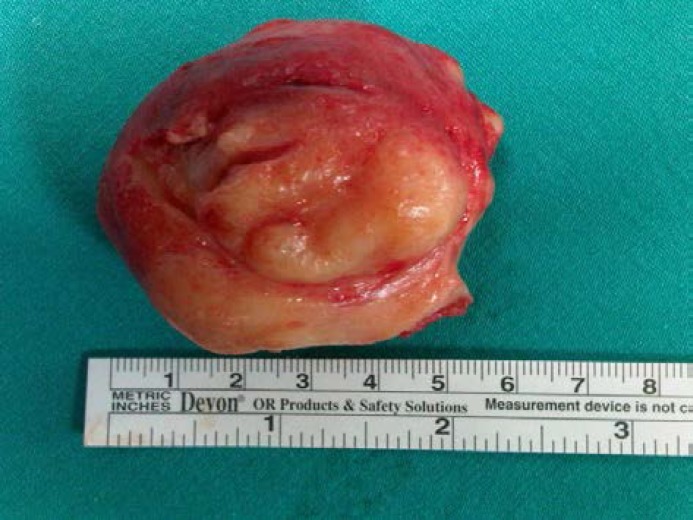
Dimension of myoma

After clamping the peduncle, 5×6 cm mass was removed. Vaginal defect was repaired with absorbable 2/0 polyglactin 910. Histopathologic evaluation confirmed leiomyoma with degeneration and inflammation (Figure 2). The postoperative course was uneventful and she was discharged without any complication on the second postoperative day. Postoperative ultrasonographic examination revealed a normal pelvic texture. 

## Discussion

Classification of submucous myomas is based on myoma within cavity degree. It is important to determine extension of submucous myoma into myometrium before surgery. According to European Society of Hysteroscopy classification system, pedunculated submucous myoma is classified as type 0 submucous fibroid. Cohen and Valle suggested that sonohysterogram is required to appreciate myometrial involvement degree (2). Because of intact hymen, we could not perform sonohysterogram for our patient. The correct diagnosis was not made until myoma could be confirmed visually.

Vaginal approach is simple and recommended modality for relatively small and prolapsed myomas. Suggested techniques include hysteroscopic resection of peduncle, non-hysterescopic cutting or twisting of peduncle and morcellation or vaporization of myoma nodule (2, 3). Golan *et al* reported successful management of cases with pedunculated myomas with vaginal myomectomy in their series (1). They concluded vaginal myomectomy is relatively short, simple, definitive, and safe method. On the other hand, hymenal integrity accounts of great importance for some individuals. For many young women, loss of virginity may result with social burden and removing such large masses outside the vagina may pose potential risk for losing virginity. 

There are several hymen types, and some of them do not bleed during first intercourse. Thus, sometimes women have to prove their virginity with vaginal examination. Some societies like Turkey, it is important to preserve hymenal integrity for possible future proof via vaginal examination. In such cases, patient desire and informed consent should be taken into consideration and myomas should be managed with reasonable diligence. Few reports have been discussed the protection of hymenal integrity during hysteroscopy so far (4-6). They underlined that hysteroscopic approach is generally successful for small pedunculated submucous myomas. Despite this clear advantage, the technique is somehow limited during the removal of large masses. In the present case, the size of the myoma was thought to outrage hymenal integrity while removing and laparotomic route was decided along with patient’s desire. In this method, it is important to remove the fibroid and base as a whole to prevent any recurrence. 

In conclusion, hysteroscopy remains as suggested option in submucous pedunculated myomas unless several factors such as myom size, anatomic obstacles or patient’s hymenal consideration limit the technique. In many traditional cultures, it is important that hymen ring is not injured during surgery so as to maintain the patients’ state of virginity. As an alternative, colpotomy and myomectomy via mini-laparotomic rout seems feasible with favorable morbidity when hymenal integrity matters. Furthermore, mini-laparotomi allows patient to feel confident during this critical intervention.

## Conflict of interest

The authors report no conflicts of interest. The authors alone are responsible for the content and writing of the paper. 
